# The Microbiota in Systemic Lupus Erythematosus: An Update on the Potential Function of Probiotics

**DOI:** 10.3389/fphar.2021.759095

**Published:** 2021-11-23

**Authors:** Xirui Guo, Xuerong Yang, Qi Li, Xiaoyan Shen, Huiyun Zhong, Yong Yang

**Affiliations:** ^1^ Department of Pharmacy, Chengdu Second People’s Hospital, Chengdu, China; ^2^ Department of Pharmacy, Sichuan Academy of Medical Sciences and Sichuan Provincial People’s Hospital, School of Medicine, University of Electronic Science and Technology of China, Chengdu, China; ^3^ Department of Pharmacy, Sichuan Vocational College of Health and Rehabilitation, Zigong, China; ^4^ Department of Pharmacy, The First People’s Hospital of Zigong, Zigong, China

**Keywords:** autoimmunity, microbiota, probiotics, systemic lupus erythematosus, inflammation

## Abstract

Systemic lupus erythematosus (SLE) is a kind of chronic diffuse connective tissue illness characterized by multisystem and multiorgan involvement, repeated recurrence and remission, and the presence of a large pool of autoantibodies in the body. Although the exact cause of SLE is not thoroughly revealed, accumulating evidence has manifested that intake of probiotics alters the composition of the gut microbiome, regulating the immunomodulatory and inflammatory response, which may be linked to the disease pathogenesis. Particularly, documented experiments demonstrated that SLE patients have remarkable changes in gut microbiota compared to healthy controls, indicating that the alteration of microbiota may be implicated in different phases of SLE. In this review, the alteration of microbiota in the development of SLE is summarized, and the mechanism of intestinal microbiota on the progression of immune and inflammatory responses in SLE is also discussed. Due to limited reports on the effects of probiotics supplementation in SLE patients, we emphasize advancements made in the last few years on the function and mechanisms of probiotics in the development of SLE animal models. Besides, we follow through literature to survey whether probiotics supplements can be an adjuvant therapy for comprehensive treatment of SLE. Research has indicated that intake of probiotics alters the composition of the gut microbiome, contributing to prevent the progression of SLE. Adjustment of the gut microbiome through probiotics supplementation seems to alleviate SLE symptoms and their cardiovascular and renal complications in animal models, marking this treatment as a potentially novel approach.

## Introduction

Systemic lupus erythematosus (SLE) is a chronic autoimmune disease, where a large pool of autoantibodies are produced, causing the immune system to attack its tissues, resulting in damage to multiple organs and systems throughout the body (Mu et al., 2015; Yacoub et al., 2018). Its main clinical features are multiple systems and organs involvement, repeated relapse and remission, and the development of a large pool of autoantibodies against double-stranded (ds) DNA (Lisnevskaia et al., 2014; Durcan et al., 2019; Fava and Petri, 2019). Individuals affected by SLE have extensive symptoms and course of the disease, the most frequent of which are fever, fatigue, facial butterfly erythema, photosensitivity, muscle or joint pain, arthritis, and renal symptoms (Goldblatt and O’Neill, 2013). Moreover, patients with SLE have an increased incidence of atherosclerosis, thrombosis, arteritis, embolization, and vascular spasm (Kasselman et al., 2018). The most lethal outcomes in SLE patients are infection, severe multiple organ injury, especially damage to the nervous system and kidney (Lee et al., 2016; Yen et al., 2017). Production of the immune response in SLE is distinguished by an overreaction of B cell and T cell responses, and impaired self-tolerance to autoantigens. (Lisnevskaia et al., 2014; Durcan et al., 2019; Fava and Petri, 2019; Kiriakidou and Ching, 2020). The prevalence of SLE varies widely from region to region, with the current global prevalence approaching or even above 50 to 241 per 100,000 adults, among which the prevalence rates of African Americans, American Indians, and Alaska Natives are higher (Helmick et al., 2008; Ferucci et al., 2014; Somers et al., 2014; Rees et al., 2017; Nikolopoulos et al., 2020). The prevalence of SLE is higher among African Americans and Europe, which is less prevalent in Africa (Symmons, 1995; Pons-Estel et al., 2010). The severity of the disease may also vary by ethnic background, with patients of African and Latin American descent commonly more severe (Carter et al., 2016). Particularly, SLE is strikingly dominated by women of childbearing age, with nearly 10 female patients for every male suffering from the disease (Carter et al., 2016; Durcan et al., 2019; Fava and Petri, 2019; Fanouriakis et al., 2021). Among women aged 15 to 44, the ratio of women to men is 13:1 while the ratio is only 2:1 between children and the elderly (Petri, 2002; Danchenko et al., 2006).

A variety of medications are applied to SLE therapy, including glucocorticoids (GCs), antimalarial agents, nonsteroidal anti-inflammatory drugs (NSAIDs), immunosuppressive agents, and B cell–targeting biologics. NSAIDs block the synthesis of prostaglandins by attenuating the enzymes cyclooxygenases (COX-1 and COX-2) to counter inflammation and pain. The adverse reactions with the highest incidence of this drug are gastrointestinal gastritis, nephrotoxicity, fluid retention (Kiriakidou and Ching, 2020). Hydroxychloroquine is the cornerstone of lupus treatment (Kiriakidou and Ching, 2020). Hydroxychloroquine or other antimalarial agents have the effect of immunomodulatory and antithrombotic (Chrisman et al., 1976; Espinola et al., 2002; Wang et al., 2019). But the drugs can induce retinopathy, skin pigmentation, and rare cases of neuromuscular or cardiac toxicity (Marmor et al., 2016). GCs are the most commonly used agents in SLE-induced remission therapy and are consistently recommended by the guidelines as first-line agents for the control of SLE. (Gordon et al., 2018; Fanouriakis et al., 2019; Kiriakidou and Ching, 2020). The side effects that may occur after taking GCs are gastrointestinal adverse reaction, metabolic disorders, infections, weight gain, hypertension, psychiatric disorders, lipodystrophy, fractures, and adrenal suppression, which are mainly dose and time-dependent (Saag et al., 1994; van Staa et al., 2002; Wei et al., 2004; Da Silva et al., 2006; Warrington and Bostwick, 2006; Dixon et al., 2011; Sarnes et al., 2011). Furthermore, immunosuppressant therapy is suggested for SLE patients who continue to respond poorly to GCs and hydroxychloroquine combination therapy, or who cannot adjust the dose of GCs to a relatively safe dose. Immunosuppressive agents such as methotrexate inhibit DNA synthesis and increase the release of adenosine, but some patients were forced to stop using the drug because of intolerance to adverse reactions (Kiriakidou and Ching, 2020). What occurs most after the application of methotrexate are gastrointestinal side effects (nausea, vomiting, diarrhea), hepatotoxicity, and blood-related toxicity (anemia, leucopenia) (Sakthiswary and Suresh, 2014; Andreoli et al., 2017). For patients with refractory (ineffective after conventional treatment) or recurrent SLE, the administration of biological agents can reduce disease activity, disease recurrence rate and reduce hormone dosage (Wei et al., 2016; Alshaiki et al., 2018). Belimumab targets B-lymphocyte stimulator inhibits B-lymphocyte proliferation and activation (van Vollenhoven et al., 2012), but its common adverse effects are hypersensitivity reaction, gastrointestinal toxicity, myalgias, depression, migraine, infection (Lee and Song, 2018; Peterknecht et al., 2018). Rituximab depletes CD20-expressing B lymphocytes, but patients may occur an infusion reaction, infection, progressive multifocal leukoencephalopathy (rare) (Alshaiki et al., 2018; Peterknecht et al., 2018). In addition, other measures can be used to treat SLE. When SLE develops to severe or refractory levels, the addition of plasma exchange or DNA immunosorbent as adjunct therapy may be considered, which may ameliorate clinical symptoms rapidly but cannot improve the outcome (Kronbichler et al., 2016). In summary, all the medicines used in the treatment of SLE induce adverse reactions, whereas, probiotics supplementation appears to have no significant side effects clinically. Hence, it is necessary to further investigate probiotics for the exploration of theoretical basis as adjuvant therapy in SLE patients.

The precise pathogenesis of SLE is not entirely revealed, it is believed to be caused by the human immune system attacking self-tissues after being abnormally activated. The pathogenesis of SLE may be related to genetic, hormones and, environmental factors (infection, drugs, UVA light) (De Luca and Shoenfeld, 2019). Nevertheless, with research on intestinal flora dysregulation going depth recently, dysbiosis as a vital internal environmental factor has also been shown to be concerned with SLE (Meng et al., 2019). In 1994, Apperloo-Renkema et al. first demonstrated experimentally that alterations in intestinal microbiota composition can cause SLE in animal models, possibly due to a weakened defense of native gut microbes against foreign bacteria (Apperloo-Renkema et al., 1994). Repeated antigen stimulation may lead to changes in intestinal microecology, confusion of the immune system, and the body subsequently attacks its tissue by producing antibodies or sensitized lymphocytes (Zhang and Reichlin, 2008). The production of these antibodies is exacerbated by extensive inflammatory responses, which leads to a range of clinical symptoms and further complications associated with SLE (Zhang and Reichlin, 2008). Long-term use of probiotics is believed to neutralize an imbalance in the gut microbiota that results in the reduced antibody production and suppressed inflammatory response, leading to attenuation of severity, signs and, manifestation of SLE (Esmaeili et al., 2017). In a study assessing the resistance of intestinal microbiota to pathogen colonization in SLE patients and healthy controls, the colonization resistance of patients with active SLE is lower than that of healthy people, which indicates that the incomplete normal intestinal microbiota may lead to more intestinal transfection of pathogenic bacteria (Apperloo-Renkema et al., 1994). The study presented above confirmed that the balance of intestinal microbiota is associated with the pathogenesis of SLE, but it is still unclear whether administering probiotics to restore normal intestinal flora and reduce inflammation may have therapeutic benefits for SLE patients or not (van der Meulen et al., 2016).

In brief, raising an understanding of how to ameliorate gut dysbiosis could help explore an alternative approach to prevent or alleviate SLE. Therefore, the alteration of microbiota associated with SLE was reviewed and the function and mechanisms of probiotics in the development of SLE animal models were also discussed.

## An Inflammatory Pathway of SLE

SLE is a multifactorial caused disease, and the pathogenesis is considered to be related to hormonal, environmental and genetic factors that lead to an intolerance to autoantigens (Rahman and Isenberg, 2008; De Luca and Shoenfeld, 2019). SLE is featured by the generation of autoantibodies, aggregation of autoreactive and inflammatory T cells, and abnormal production of inflammatory cells and pro-inflammatory cytokines (Buckner, 2010; Tsokos, 2011; Rastin et al., 2013). Autoantibodies produced by autoimmune B cells bring about the generation and accumulation of immunocomplex which do harm to multiple organs, containing the skin, joints, heart, kidneys, and brain (Zhang and Reichlin, 2008; Tsokos, 2011). Although SLE is regarded as mainly B cells mediated disease, there is some evidence indicating the significance of unbalanced regulatory T (Treg) cells in the development of SLE (Buckner, 2010; Ma et al., 2010; Talaat et al., 2015). In addition, it has been proven that improved T helper cell 17 (Th17) amount and effect play a crucial role by secreting pro-inflammatory cytokines, such as interleukin (IL)-17 and IL-23), as the primary trigger of autoimmune response, and these cytokines are related to the inflammatory formation and tissue damage in SLE (Crispín et al., 2008; Doreau et al., 2009; Chen et al., 2010; Pan et al., 2013). Studies have demonstrated that strengthening Treg cells restrain abnormal reactions of effector T cells, which can steadfastly alleviate autoimmune and inflammatory responses (Shevach, 2009; Lavi Arab et al., 2015; Reihani et al., 2015). It has been identified that patients with SLE have decreased numbers and functional deficiencies of Tregs as well as the resistance of effector T cells to the inhibitory effects of Tregs, which exert significant effects in the pathogenesis of SLE (Lyssuk et al., 2007; Valencia et al., 2007; Gómez et al., 2009; Esmaeili et al., 2017). It is reported that anti-inflammatory cytokines, such as transforming growth factor β (TGF-β) and pro-inflammatory cytokines, including IL-6, IFN-γ, and IL-23/IL-17 are drastic in every developmental stage of SLE (Su et al., 2012). Therefore, restoration of unbalanced cytokines and defective immune cells may be a potential remedial strategy for alleviating SLE manifestations (Esmaeili et al., 2017).

## The Microbiome and Probiotics

### The Gut Microbiota

The intestine contains the largest complex mic-ecosystem in humans, which can be regarded as an independent organ in the body (Van de Wiele et al., 2016). According to high-throughput culture-independent sequencing analysis, the microbiome of the gut tract is more complicated than those of other parts of the body, with over 1,000 microorganisms identified so far, and the total biomass is close to 1,000 colony-forming units 10^13^∼10^14^(CUF) (Claesson et al., 2009; Sankar et al., 2015). The intestine microbiota is dominated mainly by two phyla (approximately 90%) *Firmicutes* and *Bacteroidetes*, and the rest is involved in *Actinobacteria*, *Proteobacteria*, *Synergistetes*, *Verrucomicrobia*, *Fusobacteria*, and so on (Eckburg et al., 2005; de la Visitación et al., 2019). The existing methanogenic archaea, yeasts, and viruses (mainly phages) increase the complexity of the gut microbiota (Lozupone et al., 2012). Although only two phyla have predominance in the gut microbiota, there are striking differences in the intestinal microecology between people and people across different life cycles (Van de Wiele et al., 2016). Individual diversity in host genes, mode of delivery and lactation, geographic origin, age, diet, disease, drug uptake, and lifestyle contribute to differences in intestine microbiota composition (Ley, 2015). With the development of the functional characteristics of individual microbiota, growing evidence shows that the gut microbiota participated in critical activities related to disease and health. It has been proven that the functions of the human gut bacteria are to affect digestion, provide nutrients, form intestinal barriers and produce colonization resistance, regulate the development of intestinal epithelium, as well as to modulate the activation and progression of the immune system (Van de Wiele et al., 2016).

Therefore, any factors that disrupt the host-microbial balance (such as acute alters in dietary behavior; malnutrition; pathogen infection; inflammation; administration of anti-biological drugs; gastrointestinal surgery; etc.) may influence the homeostasis of microbiota which exerts a significant impact on the regulation of host immune functions (Ogura et al., 2003; Cho, 2008; De Filippo et al., 2010; Delzenne et al., 2011).

### Probiotics

Probiotics are living organisms that regulate the gut microbiome in various ways to improve intestinal health. Probiotics can affect immune homeostasis by keeping a healthy microbial balance, and can also adjust mucus secretion through intestinal epithelial cells, thereby contributing to maintaining the stability of the mucus barrier and providing resistance to pathogen colonization (Bron et al., 2017; de Oliveira et al., 2017). Besides, Probiotics can promote the generation of multiple nutrients such as SCFAs and vitamins, which contribute to form the entire host intestinal microbiome (Yadav et al., 2013; de Oliveira et al., 2017). Moreover, probiotics participate in the degradation of toxic compounds and the production of antimicrobial compounds, like bacteriocins (de Oliveira et al., 2017). Therefore, these probiotics can be used as a treatment option for immune-related diseases (Balakrishnan and Taneja, 2018).

Probiotics have been widely evaluated for their benefits in preventing or treating extensive diseases, including infection, inflammation, cancer, and autoimmune diseases in animal and human trails (Borchers et al., 2009). The recorded probiotic-inducing impacts include suppression of infection, immune regulation, prolonged remission of patients with ulcerative colitis, treating or preventing infective or antibiotic-associated diarrhea in both adults and infants, assisting in the eradication of Helicobacter pylori, improving nonalcoholic fatty liver disease and metabolic diseases, reducing the recurrence rate of colorectal cancer and alleviating lactose intolerance symptoms (de Vrese et al., 2001; Van Niel et al., 2002; Bengmark, 2003; Gill, 2003; Gionchetti et al., 2003; Kalliomäki et al., 2003; Tamboli et al., 2003). Probiotics have been reported to exert their beneficial effects mainly in three ways, containing competitive exclusion, antibacterial action, and regulation of immune responses. It has been found that the administration of immunoregulatory probiotics in the prevention or treatment of autoimmune diseases is mainly attributable to improving the inflammatory responses and modulating tolerance in the host to pathogens (Esmaeili et al., 2017).

### Mechanism of Action of Probiotics

It has been discovered that probiotics influence each segment of the intestine, containing the intraluminal microbiota, the epithelial microbial, mucosal barrier, the lamina propria rich in lymphocytes and plasma cells, the blood vessels and nerves of lamina propria components, the underlying smooth muscles commanding movement and the mesenteric lymph nodes associated with systemic immunity (Liu et al., 2018). In mechanism, immunomodulatory probiotics are known to prevent inflammation and modulate immunity to improve SLE symptoms (Liu et al., 2018). As Liu et al. (Liu et al., 2018) summarized that short-chain fatty acids (SCFAs) generated by *bifidobacterium*, *lactobacillus*, and symbiotic bacteria combine and activate receptors (FFAR2, FFAR3, or GPR109a) on enterocytes to inhibit inflammatory responses by blocking nuclear factor-κ-light chain enhancer of B cells activation pathway. SCFAs also suppress histone deacetylases to facilitate amassing of Tregs and discharge glucagon-like protein-1/peptide tyrosine to respond to the enteric and central nervous system, thereby affecting intestinal homeostasis and motion. They also induce tolerogenic dendritic cells (DCs), which induce immature CD4^+^ T cells to differentiate into Tregs. The above response restraint the generation of cytokines via neutrophils and macrophages by binding to receptors. Adenosine and its derivative inosine interact with adenosine receptor-2A expressed on T cells to enhance Treg effects and suppress TH1 and TH17 subsets inflammation. Histamine generated by *L. reuteri 6475* reacts on H2 receptor located in intestinal epithelial cells and macrophages to decrease the secretion of proinflammatory cytokines, containing tumor necrosis factor (TNF)-α, MCP (monocyte chemoattractant protein)-1, and IL-12. In conclusion, the pivotal metabolites generated by probiotics exhibit anti-inflammatory properties and improvement of the intestinal barrier function during the disease.

## The Microbiota Studies in SLE

Studies that described the microbiota of SLE are relatively limited, although the increasing prevalence of Crohn’s disease (CD) in patients with SLE (Shor et al., 2016) has sparked interest in its involvement. Although the pathogenesis of SLE is not completely understood, an imbalance in the microbiome has been manifested to be associated with the establishment of SLE (Hevia et al., 2014; De Luca and Shoenfeld, 2019). Until now, human studies that investigate the connection between the microbiome and SLE initiation are observational case-control studies that compare differences of the human microbiome in areas like the gut or buccal cavity between SLE patients and controls (Arron et al., 2014; Hevia et al., 2014; Zhang et al., 2015; López et al., 2016). Therefore, revealing the microbial composition and possible function of these microbes in SLE patients may illuminate the cause and development, and may even find diagnostic biomarkers.

SLE patients have compositional and functional alterations in gut microbiota, possibly due to a weakened defense of native gut microbes against foreign bacteria (Apperloo-Renkema et al., 1994). Noticeable increase of several genera, including *Rhodococcus*, *Klebsiella*, *Eggerthella*, *Prevotella*, *Eubacterium*, and *Flavonifractor*, has been found in patients with SLE, whereas, *Dialister* and *Pseudobutyrivibrio* decreased (Hevia et al., 2014; He et al., 2016; Chen et al., 2017). Quantitative polymerase chain reaction analysis confirmed that the ratio of *Firmicutes* to *Bacteroidetes* was lower in patients with SLE, and the abundance of some families of *Firmicutes* was decreased (López et al., 2016) (Hevia et al., 2014; Neuman and Koren, 2017; van der Meulen et al., 2019) (Table 1). Such alterations are also present in other diseases, such as Crohn’s disease and type 2 diabetes mellitus (Man et al., 2011), suggesting that an overall imbalanced microbiota state is not specific to SLE (Larsen et al., 2010). A similar study on the composition of gut microbiota in 45 Chinese patients with SLE was in accordance with the results mentioned above, showing lower *Firmicutes* and higher *Bacteroidetes* in SLE patients (He et al., 2016). Downregulating inflammation can be achieved in several ways, such as elimination of apoptotic cells and cell debris, clearance of oxidized lipids, and blocking the stimulation of mitogen-activated protein kinase (MAPK) and other pro-inflammatory cytokines (Grönwall et al., 2012; López et al., 2016). As the anti-dsDNA titer increased, the frequency of the Synergistetes, which was positively associated with the rate of *Firmicutes* to *Bacteroidetes* in healthy controls, verged to decrease in SLE patients and was present a significantly negative association with the level of proinflammatory cytokines IL-6 in serum, meanwhile, correlating positively with natural protective IgM anti-phosphorylcholine secreted by B1 cells (López et al., 2016).

**TABLE 1:** The shift of microbiota in SLE.

Bacteria	Changes	Mechanism of pathogenesis/potential metabolic function/other associations	Ref
*Bifidobacterium bifidum*	↑	• prevents excessive activation of CD4(+) lymphocyte	López et al. (2011); López et al. (2016)
		• generates fewer CD25^high^ cells	
		• generates IL-17, Th17, and functional Treg	
		• reduces IFNc and TNFa	
		• reverts the up regulatory effect on CD25 expression	
		• reduces the percentage of Foxp3+ cells included within the CD25^high^ population	
		• induces phenotypic DC maturation	
*Clostridia strains* ^*^	↑	• restores Th17/Th1 balance	López et al. (2016)
		• reduces the IL-17/IFNγ ratio induced by DCs	
		• reduces the IL-17/IFNγ balance	
F/B ratio	↓	• increases inflammation	Hevia et al. (2014); Neuman and Koren (2017); van der Meulen et al. (2019)
		• overrepresents oxidative phosphorylation and glycan utilization pathways	
		• some effects are species-dependent	
*Bacteroidetes*	↑	• glycan-degrading activity	Hevia et al. (2014)
		• associated mucin-degrading sulfatase activity contributing to impaired epithelial cell layer and chronic inflammation	
*Firmicutes*	↓	• produces butyrate	Hevia et al. (2014)
*Prevotella spp.*	↑	• considered a gut commensal associated with a fiber-rich diet	He et al. (2016); Ley (2016); Chen et al. (2017)
		• SCFA producer	
*Eggerthella (Coriobacteriaceae)*	↑	• Not well characterized yet	Gardiner et al. (2015); He et al. (2016)
		• believed to be an emerging pathogen	

Abbreviations: F/B, *Firmicutes/Bacteroidetes*; TH, T helper; DC, Dendritic Cells; IL, interleukin; IFN, interferon; Foxp3+, forkhead Box P3+; ↓, decrease; ↑, increase; *, Two mixed strains of clostridium (*Ruminococcus obeum DSM25238* and *Blautia coccoides DSM935*).

In female SLE patients, studies have shown the abundances of *Lactobacillaceae* decreases, while the levels of *Lachnospiraceae* increase, both of which belong to the *Firmicutes phylum* (Zhang et al., 2014; Mu et al., 2015; Neuman and Koren, 2017). Notably, comthe level of abutyrate-producing bacterium *Lachnospiraceae* was augmenter in SLE patients compared with healthy controls, thus *Lachnospiraceae* or any bacterium that produces butyrate may be unable to inhibit inflammation in SLE cases (Kakiyama et al., 2013; Zhang et al., 2014; Kasselman et al., 2018; Luo et al., 2018). Further, patients with SLE tend to generate more CD25^high^ cells, whereas *Bifidobacterium bifidum (B. bifidum)* strain can revert to the up regulatory effect (López et al., 2016). On the other hand, the microbiota isolated from the feces of SLE patients has been found to accelerate the activation of lymphocytes and the differentiation of Th17 from primitive CD4^+^ lymphocytes (López et al., 2016). Additionally, *B. bifidum* may prevent lymphocyte activation whereas mixtures of two Clostridia strains supplementation, including *Ruminococcus obeum DSM25238* and *Blautia coccoides DSM935*, restore Th17/Th1 balance (Atarashi et al., 2011; Atarashi et al., 2013; López et al., 2016). Alternatively, increased numbers of *Selenomonas*, *Veillonella*, *T. denticola*, and *Leptotrichia* are directly associated with raised concentrations of inflammatory factors like IL-6, IL-17, and IL-33, which are indicative of a decline in oral microbial species diversity in SLE patients (Corrêa et al., 2017).

## Roles of Probiotics Against SLE

As a result of the above-mentioned findings, researchers believe that SLE treatment with probiotics (Table 2), has already presented some benefits like in other autoimmune diseases, can help ameliorate the symptomatology of disease (Schiffer et al., 2011; Zamani et al., 2016). Studies in animal and human trials have identified the potential benefits of probiotics in the alleviation and suppression of inflammation and autoimmune responses (Liu et al., 2018). Research in the SLE animal model has demonstrated that certain probiotic strains, including *B. bifidum*, *Lactobacillus*, *Ruminococcus obeum*, and *Blautia coccoides*, contribute to regulating excessive inflammation and restore tolerances (Esmaeili et al., 2017).

**TABLE 2:** Effects of probiotics in SLE animal models.

Probiotic strain	Model	Effects	Ref.
*Lactobacillus strains* ^*^	MRL/lpr, mice	• suppresses the generation of IL-6 IL-10	Mu et al. (2017)
		• decreases the production and renal deposition of pathogenic IgG2a	
		• suppresses pathogenic Th 17 cells	
		• increase Treg cells	
		• rebalances T cell subsets in the kidney	
*Lactobacillus*	Female pristane-induced BALB/c mice	• decreases IL-6	Mardani et al. (2018); Khorasani et al., 2019)
		• reduces Th1–Th17 polarization	
		• reduces the number of Th17 cells	
		• decreases the expression of IL-17 mRNA and IL-17 protein levels	
		• decreases the level of anti-dsDNA, ANA, anti-RAP	
		• enhances Tregs and the expression level of FoxP3	
*Lactobacillus reuteri GMNL 263*	NZB/W F1	• decreases TLR-4, TLR-5, TLR-7, and TLR-9	Hsu et al. (2017); Tzang et al. (2017)
		• declines the generation of IL-1β, IL-6, and TNF-α	
		• increases the differentiation of CD4^+^CD25 + FoxP3+ T cells	
		• increases the proportion of CD4^+^CD25 ^+^ T cells in CD4+T cells	
		• increases the mRNA level of Foxp3 in CD4^+^CD25 + T cells	
*Lactobacillus paracasei GMNL 32*	NZB/W F1	• reduces the expression of IL-1β, IL-6, and TNF-α	Hsu et al. (2017); Hu et al. (2017); Tzang et al. (2017)
		• down-regulates TLR-4, TLR-5, TLR-7, and TLR-9	
		• increases heart weight and the ventricular wall thickness	
*Heat-Killed Lactobacillus reuteri GMNL-263*	NZB/W F1	• reduces TUNEL-positive cells, Fas death receptor-related elements, and TNF-R1	Yeh et al. (2021)
		• increases the levels of survival protein phospho-AKT	
		• decreases MMP-9 and the levels of MMP-9 proteins	
*Lactobacillus fermentum CECT5716*	NZB/W F1	• reduces the elevated T, B, Treg, and Th-1 cells in mesenteric lymph nodes	Toral et al. (2019); de la Visitación et al. (2020)
		• decreases the plasma levels of IL-17a, IL-10, IFN-g, TNF-a, and IL-2 to normal levels	
		• decreases gene expression of IL-6, IL-β, THF-α, and TLR-4 in the aorta	
*Bacteroides fragilis ATCC 25285*	Female C57BL/6J and B6.MRL-Faslpr/J lupus-prone mice	• reduces the production of TNF-α, IL-6, and MCP-1	Li et al. (2020)
		• promotes CD1d production in B cells by Est-1 pathway	
		• inhibits CD86 expression to repair the immune response of B cells	
		• decreases the level of anti-dsDNA, total IgM, total IgG, BUN, Cre, and RBP in serum	
		• increases CD1d expression level	

Abbreviations: IL, interleukin; Th, T-helper; Treg, regulatory T cells; Foxp3, forkhead Box P3; ANA; TNF, tumor necrosis factor; TLR, toll-like receptor; TNF, tumor necrosis factor; TUNEL, terminal deoxynucleotidyl transferase, 2′-deoxyuridine, 5′-triphosphate (dUTP)-mediated nick-end labeling; MMP-9, matrix metallopeptidase 9; MCP, monocyte chemoattractant protein-1; BUN, blood urea nitrogen; *, A mixture of 5 Lactobacillus strains (*L. oris, L. rhamnosus, L. reuteri, LactobaCallus johnsonii*, and *L. gasseri*).

Researchers found that enteral administration of combinations of *lactobacilli* or *L. reuteri* alone in MRL/LPR mice, SLE mouse models, can skew the balance of Treg–Th17 toward Treg cell advantage in the kidneys, reduce endotoxemia, decrease the concentrations of dsDNA-reactive IgG, decrease urinary protein, and ameliorate the survival rate of patients (Mu et al., 2017). These outcomes were related to a shift in intestinal microbiota and extension of *Lactobacilli*, *Clostridiales*, and *Desulfovibrionales*. Mu et al. (2017) found that *Lactobacillus spp.* supplementation plays an anti-inflammatory role through reducing IL-6 and enhancing IL-10 generation in the intestine. The supplement of therapeutic *Lactobacillus* enhanced circulating IL-10 and declined IgG2a, which is regarded as a main immune deposition in MRL/lpr mice kidney. These benefits were observed in female and unsexed male mice, rather than in male functional mice, indicating that intestinal flora may regulate inflammation in a sex hormone-dependent pattern. According to Mardani et al.(2018), the consumption of *Lactobacillus delbrueckii* and *actobacillus rhamnosusin* to Female pristane-induced BALB/c mice improved the symptoms of SLE, exhibit anti-inflammatory properties by attenuating the generation of Th7 and down-regulating its major cytokines of IL-17a, one of the critical mediators in the formation and progression of inflammation.

*L. reuteri GMNL 263* can down-regulate cytokine levels and repaired Tregs in NZB/W F1 mouse model, which is distinguished by oxidative stress and reduction of regulatory Tregs levels in circulation (Tzang et al., 2017; Liu et al., 2018). Alternatively, *L. GMNL 263(GMNL 263)* showed a diverse mechanism in the NZB/W F1mouse model of SLE (Tzang et al., 2017). These probiotics strains can enhance the production of Treg lymphocytes and the levels of transcription factor fork head box P3 (FoxP3), which is the hallmark of a natural Treg. These cells are in charge of regulating these pro-inflammatory lymphocytes and have significant anti-inflammatory characteristics. Besides, TLR-4, TLR-5, TLR-7, and TLR-9, which are the common pathogen-associated molecular pattern receptors that mediate the inflammation progression in the liver, were decreased and the antioxidant activity was increased under probiotics treatment (Hsu et al., 2017; Tzang et al., 2017). In addition, *GMNL 263* also promoted the differentiation of CD4+CD25+FoxP3+ T cells and the proportion of CD4+CD25+ T cells number in CD4+T cells of spleen and enhanced the expression of Foxp3 mRNA in CD4^+^CD25+ T cells (Hsu et al., 2017).

In similar trials, the above alterations of TLRs and oxidative stress were also found using probiotics *L. paracasei GMNL 32(GMNL-32)* and *L. reuteri GMNL 89*, although *GMNL 263* presented an effect on Treg expression in those cases (Hsu et al., 2017). The SLE-associated inflammation was also decreased with the administration of these probiotics, through enhancing the activity of antioxidation in serum and levels of CD4^+^CD25 ^+^ regulatory T cells in NZB/W F1 mice (Tzang et al., 2017). Moreover, in the treatment of these three probiotics strains, pro-inflammatory cytokines IL-1β, TNF-α, and IL-6 declined in the liver by inhibiting nuclear factor kB (NF-κB) and the signaling pathway of mitogen-activated protein kinase (Hsu et al., 2017; Hu et al., 2017). Specifically, *GMNL-32* supplement attenuated left ventricular hypertrophy and the cardiac cell apoptosis in this genetic model of lupus (Hu et al., 2017; Tzang et al., 2017). These results indicated that oral supplement of several probiotic strains, such as *GMNL32*, *L. reuteri GMNL-89*, and *L. reuteri GMNL263*, to NZB/W F1 mice can not only mitigates hepatic inflammation and apoptosis caused by SLE, but also presents a protective function on cardiac cells of lupus-prone mice (Hsu et al., 2017; Hu et al., 2017; Tzang et al., 2017).

Yeh et al. (2021) first revealed the preventive effect of *Heat-killed L. reuteri GMNL-263* on expanded interstitial spaces and abnormal myocardial structures in the hearts of NZB/W F1 mice, and lowered area of fibrosis and rescues cardiomyocyte arrangement, which demonstrate the clinical applications of the *Lactobacillus* in SLE-related cardiovascular diseases therapy. Because *Heat-Killed L. reuteri GMNL263* prevented the development of the proinflammatory response, cardiac and renal hypertrophy complications in SLE were averted (Yeh et al., 2021). Compared with the controls, the anti-apoptotic effects were observed in the NZB/W F1 mice, and the significant declines of TUNEL-positive cells, Fas death receptor-related elements, and apoptosis were also detected after the consumption of *GMNL-263*. Additionally, administration of *L. reuteri GMNL-263* to NZB/W F1mice present markedly higher levels of phospho-AKT (survival protein) than in NZB/W F1 control group. In fact, feeding *L. Paracasei GMNL 32* was also detected to exhibit a similar protective pathway and prevent cardiac complications associated with SLE in NZB/W F1 mice. The Heat-killed *L. reuteri GMNL-263* was a kind of dead bacteria, which was killed after heat treatment at 121°C for 5 min in 0.9% sterile NaCl and were made into powder freeze-dried. During the experiment, the powder was dissolved into a probiotic solution and fed to mice. Live probiotics provide barrier protection and immune system modulation; while components of dead cells exert an anti-inflammatory response in the gastrointestinal tract. Both live and dead probiotics can exert specific actions. To sum up, both *L. reuteri GMNL-263* and *L. paracasei GMNL 32* have been observed to exert cardioprotective properties by reducing TNF-R1, Fas-associated protein with death domain (FADD) and fibrosis proteins matrix metallopeptidase 9 (MMP-9).

Research has found *L. fermentum CECT5716(LC40)* protects the kidney and cardiovascular complications as well as disease activity in a female mouse model of SLE (Toral et al., 2019; de la Visitación et al., 2020). The administration of the immune-modulatory bacterium LC40 could increase the number of Bifidobacterium in the intestine of female NZB/WF1 mice. *LC40* can reduce the activity of lupus and splenomegaly in SLE mice, improving the integrity of the intestinal barrier, reducing the plasma level of lipopolysaccharide (LPS), and subsequently decreasing the immune activation, which was characterized by reduced T and B cells in mesenteric lymph nodes (MLNs) and declined plasma pro-inflammatory factors, containing TNF-α, IFN-γ, IL-17a, and IL-21. Since probiotics prevented the progression of proinflammatory responses, complications related to SLE, such as cardiac and renal hyperplasia, were prevented (Toral et al., 2019). Another research reported that treatment with *LC40* decreased the enhanced plasma anti-dsDNA, endotoxemia, and hypertension in NZB/WF1 mice. Meanwhile, *LC40* also protected lupus mice from deterioration in renal function and kidney damage, as well as suppressing immune-complex deposition and inflammatory infiltration in glomerular, tubulointerstitial, and vascular lesions (de la Visitación et al., 2020).

In one recent experiment conducted by Li et al. (2020), oral supplementation of *Bacteroides fragilis (B. fragilis) ATCC 25285* reduced autoantibodies levels and symptoms of lupus nephritis in MRL/lpr mice. The results confirmed that *B. fragilis ATCC 25285* could improve the expression CD1d in B cells through Est-1 pathway, but suppress the expression of CD86 through SHP-2 signaling pathway to restore the immune response of B cells. Furthermore, levels of anti-dsDNA, total IgG and total IgM, as well levels of BUN, CRE, and RBP in serum decreased in MRL/lpr mice. In parallel, *B. fragilis ATCC 25285* was found to play a role in restoring the balance of Th17/Treg in MRL/lpr mice, as it does in other autoimmune diseases (Li et al., 2020).

Through the above research, we have an overall understanding of the beneficial role of probiotics in adjuvant therapy of SLE, particularly the regulatory function of Treg and Th17 (de la Visitación et al., 2019). Dendritic and T Treg cells, cytokines like IL-6, IFN-γ, IL-17, and IL-23 are currently regarded as the most dominant mediators of dysregulation in the tolerated condition (Esmaeili et al., 2017). Different strains of probiotics may exhibit different beneficial functions but still fall within the same species. In this regard, live versus heat-killed probiotics present different properties, such as *L. reuteri GMNL-263* and *Heat-killed L. reuteri GMNL-263* (Adams, 2010). Therefore, further exploration of the potential mechanisms of probiotics is necessary, which will not only contribute to the cause and progression of SLE but may also support an alternative strategy for the comprehensive treatment of SLE, such as renal, cardiovascular, and hepatic complications.

## Discussion

It is well known that there is a connection between microbes and autoimmune diseases. Alterations of the microbiome, namely “dysbiosis” can cause autoimmune disease influenced by the factors of certain genetic backgrounds and environments (De Luca and Shoenfeld, 2019). Dysbiosis can occur with the following three situations: reduction of beneficial microorganisms, overgrowth of potentially harmful microorganisms, and decrease of microbial diversity (DeGruttola et al., 2016). In order to ameliorate the adverse effects produced by microbial imbalances during the course of the disease, it may be possible to reestablish a healthy microbiota by supplement multiple probiotic strains, such as *Bifidobacteriaum spp.*, *Lactobacillus spp.*, *Lactococcus spp.*, *Pediococcus spp.*, or more varieties (Solis et al., 2002; Homayouni et al., 2014). Furthermore, fecal microbiota transplantation (FMT) is the transplantation of healthy fecal fluids into the gut of the recipient to restore a stable intestinal flora, which affects both the endogenous and host microbes (Gough et al., 2011).

Theoretical risks of probiotics have been illustrated in case reports, including systemic infections, harmful metabolic activities, gene transfer, extreme immune activation in susceptible populations, and gastrointestinal adverse reactions (Doron and Snydman, 2015). Notably, the most frequently reported single event is fungemia caused by consumption of *Lactobacillus acidophilus* and *Lactobacillus casei* (Barton et al., 2001; De Groote et al., 2005; Ledoux et al., 2006; Vahabnezhad et al., 2013). Meanwhile, incidents of endocarditis caused by both *Lactobacillus* and *Streptococcus* probiotics have also been reported (Mackay et al., 1999; Doron and Snydman, 2015). Although probiotics supplements appear to have no clinically significant side effects, the administration of probiotics in susceptible individuals should be treated with caution.

In conclusion, the existing evidence manifests that some probiotics, such as *Lactobacillus*, which can restore dysbiosis and enhance intestinal barrier function may prevent the occurrence of cardiovascular and renal complications of SLE and alleviate its symptoms. The mechanism of intestinal microflora imbalance inducing the occurrence and development of SLE may be associated with the abnormal T cell subsets, particularly the abnormal levels of Naïve CD4+T, γδT, Tfh, Treg, and Th17 cells. Further exploration of the mechanism by which the probiotics influence the disease state of SLE, most likely through inflammation and the immune system, may contribute to the progression of future clinical treatments. Therefore, it is significant to shed light on the variation of intestine microbiota to exhibit anti-inflammatory properties, and potentially they can be considered as biomarkers to reflecting disease status. Particularly important is that more animal trials combined with clinical studies are needed to further elucidate the mechanisms for the effect of probiotics, meanwhile, to unravel whether specific probiotics bacteria have a positive impact on the treatment or prevention of SLE to develop novel therapeutic targets.
